# Clinical and Radiographic Assessment of Secondary Bone Graft Outcomes in Cleft Lip and Palate Patients

**DOI:** 10.1155/2014/231795

**Published:** 2014-11-10

**Authors:** W. Khalil, C. R. de Musis, L. E. R. Volpato, K. A. Veiga, E. M. M. Vieira, A. M. Aranha

**Affiliations:** ^1^School of Dentistry, University of Cuiabá, Cuiabá, MT, Brazil; ^2^Post-Graduation Program in Environmental Sciences, University of Cuiabá, Cuiabá, MT, Brazil; ^3^Post-Graduation Program in Dental Science, School of Dentistry, University of Cuiabá, Rua Estevão de Mendonça 1295, Apartment 2401, Quilombo, 78043-407 Cuiabá, MT, Brazil; ^4^Dental Science Program, School of Dentistry, University of Cuiabá, Cuiabá, MT, Brazil

## Abstract

*Purpose.* To compare the results of secondary alveolar bone grafts in patients with complete cleft lip and cleft lip and palate using 2 radiographic scales and according to the rate of canine eruption through the newly formed bone. *Materials and Methods.* We analyzed pre- and postoperative radiographs of 36 patients for the amount of bone in the cleft site according to the Bergland and Chelsea scales. The associations between the variables and the correlation between the scales were measured. *Results.* A total of 54.2% and 20.8% of cases were classified as type I and type II, respectively, using the Bergland scale, whereas 50% and 22.5% were classified as types A and C, respectively, using the Chelsea scale. A positive correlation between the 2 scales was observed. In 33.3% of males, 58.3% of females, 54.5% of unilateral cleft cases, and 12.5% of bilateral cleft cases, the permanent canines had erupted. Bone grafts performed prior to canine eruption achieved more satisfactory results. *Conclusions.* Our results suggest that both radiographic scales are important tools for the evaluation of bone grafts. Additionally, longer time periods of evaluation were associated with improved results for patients with secondary alveolar bone grafts.

## 1. Introduction

Among the congenital craniofacial anomalies, cleft lip and palate (CLP) represent the second most common orofacial malformation in live births [1]. In Brazil, 1 in 700 live births presents CLP [2] with a ratio of 1.6 males to every female [3].

The surgical protocol for the rehabilitation of CLP is controversial, with no consensus regarding the timing and techniques used for each stage of the reconstruction [4]. However, reconstruction of the alveolar process through an alveolar bone graft is generally approved for the treatment of CLP patients with involvement of the alveolar ridge. This treatment provides bone growth in the cleft area as well as other benefits, including the eruption of permanent teeth, posterior dental prosthetic rehabilitation, support and stability to the wing of the nose, oronasal communication occlusion (improving nasal emission and phonetics), orthodontic movement, and the insertion of dental implants [5–7]. According to the time at which the alveolar bone graft is performed, the graft is classified as primary, when it occurs early in life; secondary, when it is placed in the mixed dentition before or after eruption of the canines; or tertiary, when it is placed in the permanent dentition [8].

Bone grafts are a necessary component of the care protocol for individuals with CLP and alveolar ridge involvement. However, this method presents high rates of complications such as resorption of the grafted bone, suture dehiscence, soft tissue necrosis (especially of the palate), and graft contamination [8]. As a result, different methods for the clinical evaluation of secondary alveolar bone grafts have been proposed, including assessment of the level of periodontal insertion, eruption of the permanent canine through the cleft, and aesthetic results [9, 10]. Moreover, radiographic assessment seems to be effective and superior to clinical methods [9–11].

Based on the evidence indicating potential bone graft losses, the aim of the present study was to investigate and compare the efficacy of autogenous bone grafts in the secondary alveolar region of individuals with CLP using 2 radiographic scales, as well as to observe the eruption of the permanent canine through the newly formed bone.

## 2. Materials and Methods

### 2.1. Study Population

In the present study, 125 out of 709 patients who were treated at the Cleft Lip Palate Service by the Oral and Maxillofacial Surgery staff at the University General Hospital (Cuiabá, MT, Brazil) were selected during the period from December 2004 to February 2012. All selected patients had cleft lip or cleft lip and palate involving alveolar ridge. The selected patients presented complete unilateral cleft lip (CUCL), complete bilateral cleft lip (CBCL), unilateral cleft lip and palate (UCLP), and bilateral cleft lip and palate (BCLP). All patients with UCLP and BCLP underwent rapid maxillary expansion due to the presence of posterior crossbite and/or maxillary atresia [4]. Additionally, we selected panoramic, occlusal, and periapical radiographs of these patients for analysis.

Individuals of both genders who were in the stage of mixed dentition before or after eruption of the permanent canines, patients who underwent primary surgeries (cheiloplasty and palatoplasty), patients submitted to alveoloplasty and autogenous bone graft, and patients with standardized radiographs with satisfactory image quality were included in the sample. However, patients suffering from systemic or associated syndromes and patients with damaged or missing radiographic material were excluded.

Thus, the clinical records of 36 individuals aged between 8 and 13 years were included in the sample, resulting in a total of 40 autogenous bone grafts for evaluation.

This study was submitted and approved by the Ethics Committee in Research, CEP/UNIC (2012-008).

### 2.2. Evaluation of Clinical Records

From individuals included in the sample, we collected information on sex, age at the time of bone graft, period of the eruption of permanent canines, cleft type, donor area, period of the postgrafting radiograph, postoperative complications, and need to repeat the bone graft.

### 2.3. Clinical and Radiographic Assessment of Secondary Bone Grafts

In this study, the clinical indicator for the success of autogenous bone grafts was the eruption of the permanent canine adjacent to the cleft, which was considered erupted or nonerupted [5, 9, 11–13].

For the radiograph analysis of secondary bone grafts, a single examiner, who was previously calibrated, assessed the periapical, panoramic, and occlusal radiographs using the indicators of surgical success described by Bergland et al. [14] and Chelsea [15].

In the Bergland scale, for the assessment of bone grafts, the permanent canine adjacent to the cleft must be erupted. Thus, 29 of the 40 grafts selected were evaluated using this scale. The height of the interdental septum was observed and classified into 4 categories: type I: height of the interdental septum close to normality (<25% of bone resorption); type II: height of the interdental septum equal to or greater than 3/4 of the normal height (25% ≤ bone resorption < 50%); type III: height of the interdental septum less than 3/4 of the normal height (50% ≤ bone resorption < 75%); and type IV: bone graft failure with no continuous bony bridge across the cleft (bone resorption ≥75%).

To evaluate the success rate of the radiographic bone graft using the Chelsea scale, the position of the bone tissue in relation to the teeth adjacent to the cleft was analyzed by separating the radiographic images (40 bone grafts) into 6 categories: type A: the presence of bone tissue at the cementoenamel junction of the teeth adjacent to the cleft and at least 75% of both roots covered by bone; type B: the presence of bone tissue at the cementoenamel junction of the teeth adjacent to the cleft and at least 25% of both roots covered by bone; type C: the presence of bone tissue surrounding at least 75% of the roots in the cleft area with an apical direction; type D: the presence of bone tissue surrounding at least 50% of both roots in the cleft area with an apical to coronal direction; type E: the presence of bone tissue bridge in an area of the cleft, except in the apical and coronal directions; and type F: the presence of 25% or less of bone tissue in both roots in the apical direction.

Bone grafts of types I and II according to the Bergland scale and bone grafts of types A and C according to the Chelsea scale were considered satisfactory, whereas the other types were considered unsatisfactory [14, 15].

### 2.4. Statistical Treatment of the Results

Cohen's Kappa reliability test was performed to assess intraexaminer calibration using 2 radiographic assessments with an interval period of 30 days between them (*κ* = 0.811; *P* = 0.000).

The Mann-Whitney test was used to analyze possible associations between the types of clefts, period of eruption of permanent canine, and success rates obtained from the scale applied. To analyze the correlation between the Bergland and Chelsea scales, the age of the patients, and the periods of radiographs before and after grafting, we used the Kendall rank correlation coefficient. *P* values <0.05 were considered significant.

## 3. Results

In the characterization of the study sample (Table 1), a male predominance and a prevalence of left UCLP were observed. The autogenous bone grafts were performed predominantly in individuals younger than 10 years of age (52.7%), and most grafts were derived from the iliac crest bone (80%) and were carried out before the eruption of the permanent canine adjacent to the cleft. Few postoperative complications were observed, such as infection of the surgical area, primary graft resorption requiring repetition, and exposure of bone tissue due to suture dehiscence.

**Figure 1:**
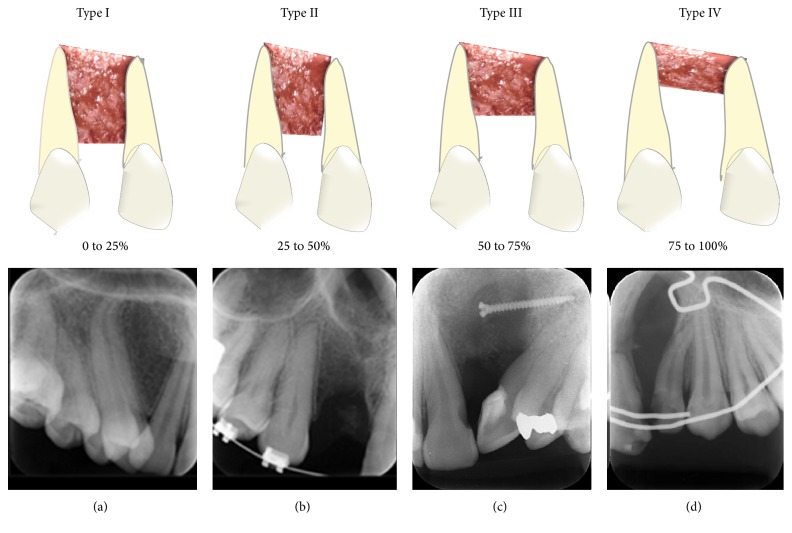
Periapical radiographic images of autogenous bone grafts according to the Bergland classification. (a) Type I: 0 to 25% of bone resorption. (b) Type II: 25 to 50% of bone resorption. (c) Type III: 50 to 75% of bone resorption. (d) Type IV: 75 to 100% of bone resorption with no continuous bony bridge through the cleft.

**Figure 2:**
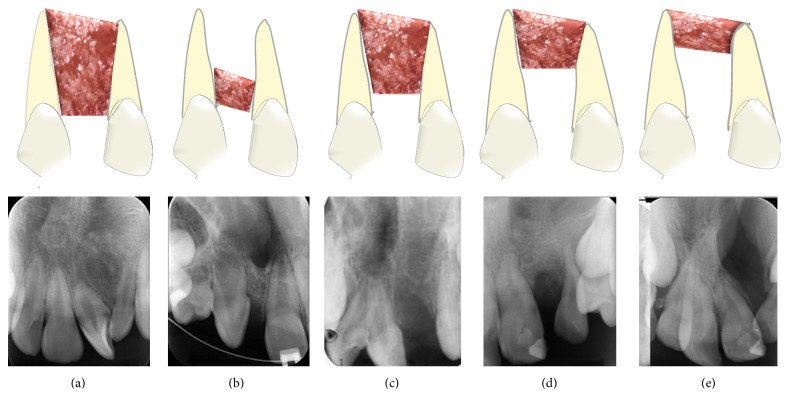
Periapical radiographic images of autogenous bone grafts according to the Chelsea classification. (a) Type A: the presence of bone tissue at the cementoenamel junction of the teeth adjacent to the cleft and at least 75% of both roots covered by bone. (b) Type B: the presence of bone tissue at the cementoenamel junction of the teeth adjacent to the cleft and at least 25% of both roots covered by bone. (c) Type C: the presence of bone tissue surrounding at least 75% of the roots in the cleft area with an apical direction. (d) Type D: the presence of bone tissue surrounding at least 50% of both roots in the cleft area, with an apical to coronal direction. (e) Type F: the presence of 25% or less of bone tissue in both roots in the apical direction.

According to the Bergland scale, the secondary autogenous bone grafts were classified as type I (54.1%), type II (20.8%), type III (16.6%), or type IV (8.3%). According to the Chelsea scale, the grafts were assessed as type A (50%), type B (2.5%), type C (22.5%), type D (12.5%), or type F (12.5%). Representative images of grafts classified using the Bergland and Chelsea scales are shown in Figures 1(a)–1(d) and 2(a)–2(e), respectively.

Because autogenous bone grafts of types I and II from the Bergland scale and types A and C from the Chelsea scale were considered satisfactory, the success rates of autogenous bone grafts were 86,2% and 82,7%, respectively. A strong correlation between the Bergland and Chelsea scales was observed (*P* < 0.01; Table 2), as well as a negative correlation (*κ* = −2.59; *P* = 0.039) between the period of post-graft radiograph and the classification of bone grafts using the Chelsea scale. This result indicated that longer postoperative assessment times (in months) were associated with improved results of the bone graft.

Associations between the effectiveness of bone grafts, as analyzed using the Bergland and Chelsea scales, and variables such as the type of cleft, laterality, side of the cleft, and period of eruption of the permanent canine were assessed. We observed a strong tendency for the association between the bone graft, before the eruption of the permanent canine, and satisfactory results of surgical procedures according to the Chelsea scale (types A and C: 82,7%; 24 satisfactory grafts out of 29 grafts performed before eruption of the canine; the other 11 grafts were performed after eruption of the canine).

Approximately 44.8% of the permanent canine teeth adjacent to the cleft erupted after the autogenous bone graft was performed (13 erupted canines out of 29 canines that were still impacted at the time of surgery), and a larger range of postoperative assessment was associated with a greater number of erupted canines (*P* = 0.001). Additionally, we observed a positive association between the number of erupted canines, the unilateral cleft, and female sex (Table 3).

## 4. Discussion

Despite the controversies regarding the optimal period for completion of bone grafts [5, 11, 15–19] and the origin and type of the bone grafting material [20], the objectives of alveolar defect repair are well defined.

Several variables can influence the selection of the ideal donor area in cases of autogenous bone grafts, including the cleft size, the bone volume required, the need for tooth eruption through the cleft, the conditions of the donor area, and the repair capacity of the individual [8, 21–23]. In this study, 80% of the grafts were derived from the anterior region of the iliac crest, due to the need for increased bone volume. Obtaining bone from this region represents a routine method for the correction of alveolar bone defects, demonstrating superior results compared to grafts from the symphysis menti [21] and cranial calvarium [8]. There is also evidence that bone grafts derived from the iliac crest require less surgical time and are associated with a shorter hospital stay for the patient and lower morbidity of the donor area [21, 22]. Additionally, the collection of bone from the iliac crest is well tolerated by patients and demonstrates favorable wound healing and aesthetics [21, 22]. Although the anterior region of the iliac crest has shown excellent results [22], Abramowicz et al. [23], it was observed that obtaining bone from the posterior region results in less bleeding and requires a shorter hospitalization time. However, posterior grafting requires a higher bone volume and increased surgical time.

Due to the reduced need for bone volume in the current study, 20% of the grafts analyzed were obtained from the intraoral region. This approach represents a safe and successful procedure that can provide shorter operating times without the need for hospitalization and the absence of morbidity in the donor area, and this procedure is also better accepted by patients [24]. Moreover, the donor area was not found to influence the effectiveness of the bone grafts, which is in agreement with a previous study [19].

Previous evidence indicates that primary bone grafts, when performed in the deciduous dentition, interfere with the growth of the anterior and inferior maxilla, thereby increasing the risk of crossbites and undermining the angles formed by the teeth and premaxilla [25, 26]. Late or tertiary bone grafts performed in adults still have potential surgical success, although less than that observed in adolescents in the mixed dentition [5, 8, 27]. In the present study, secondary autogenous bone grafts were analyzed before and after the eruption of the permanent canines, and these procedures were considered satisfactory in 82.7% and 45.4% of cases, respectively. Numerous studies have shown the increased effectiveness of secondary bone grafts performed before the eruption of the canines, which provide excellent periodontal support for teeth adjacent to the cleft; in addition, this approach favors the subsequent eruption of the canines and orthodontic alignment and ensures a minimum impediment of facial growth [5, 9, 11, 18, 19]. However, we only observed a trend towards a significant association between the time of completion of the grafts and the effectiveness of the procedure (*P* = 0.07), which may be explained by the small sample size.

The success rates of secondary bone grafts in the present study were 86,2% (Bergland scale) and 82,7% (Chelsea scale), which is consistent with earlier investigations reporting rates of 70.3% to 86% [5, 8, 11, 14, 17, 18, 27]. Moreover, these rates were shown to increase, reaching values up to 98%, for grafts performed prior to eruption of the permanent canines adjacent to the cleft [5, 11, 18, 19].

Different imaging methods have been used for the assessment of bone grafts in the alveolar region, including radiographic methods [15, 27–29], computed tomography (CT) [25, 30, 31] and ultrasound [32]. Rosenstein et al. [25] showed that the overall assessment of alveolar bone grafts using radiographic images was equivalent to that using CT. However, when analyzing each image individually, these authors observed that the radiographs overestimated (21.4%) or underestimated (17.7%) the bone volume on the root surfaces. Likewise, evidence suggests that CT may be a superior method to the use of conventional radiographs, as the 3-dimensional image can clearly identify bony bridge formation after grafting and the amount of bone at the receptor site, according to the bone cross-sectional image preview in the buccal-palatal direction [30, 31].

When occlusal radiographs were compared to periapical ones, few differences were observed, suggesting the absence of superiority of any single type for the assessment of bone grafts [16]. In this investigation, periapical, occlusal, and/or panoramic radiographs were used for the assessment of bone grafts because this retrospective study was based on the collection of data from the clinical records of selected patients, and CT is not routinely performed at the analyzed hospital institution.

The Bergland and Chelsea scales were used in this study to investigate the results of secondary bone grafts in the alveolar region. The Bergland scale represents a 4-point semiquantitative radiograph scale, which measures the height of the postgraft interdental bone septum. This scale is considered the gold standard method for analysis [14] and has been widely used [11, 16, 18, 19]. However, when identifying a bone defect in the apical root, but with normal height of the interdental bone, there may be difficulties using the Bergland assessment criteria, as the presence of cases with partial failures may be classified as successful. Thus, the Chelsea scale was developed by Witherow et al. [15] to describe a grid appearance of bone formation through the cleft, and this method can be used to identify the exact position of the bone in the cleft in relation to the root surfaces of the teeth adjacent to the cleft. To complement the Bergland scale, Trindade-Suedam et al. [27] included a few modifications by classifying grafts as either E (excellent; normal height of the interdental septum), G (good; bony septum with minimal disability), R (regular; graft with enough bone for the canine eruption, but tooth movement is deficient or a marginal defect greater than 25% of root length is present), B (bad; bone deficiency in the nasal aspect prevents tooth movement), or F (failure; complete resorption of the bone graft).

A positive correlation was observed in our study between the Bergland and Chelsea scales, in agreement with previous studies [11, 16, 19]. According to the Bergland scale, grafts are assessed after the eruption of the permanent canines adjacent to the cleft. Thus, 60% of the cases were assessed in our study, resulting in a success rate of 86,2%, which is similar to that observed in 80–96% of previous studies [11, 14–16]. On the contrary, according to the criteria of the Chelsea scale, the satisfactory result rate of 82.7% was superior to the 65% reported by Witherow et al. [15] and the 71% reported by Nightingale et al. [16] but was similar to the rate of 86% observed by Trindade et al. [11]. These differences are most likely due to differences in time after completion of the bone grafts [19, 30, 31], the width of the cleft [33], the experience of the surgeons, and the bony volume of presurgical support in the region of mesial and distal teeth adjacent to the cleft [34], among other factors.

In our research, the radiographs were performed over a period that ranged from 3 to 48 months after bone grafting. We observed a significant correlation between the postsurgical period and patient success rate. Specifically, longer assessment times of the grafts (from 25 months) were associated with improved patient results. Likewise, Toscano et al. [19] reported overall success rates of alveolar bone grafts of 70.4% in the first year of follow-up and 91.8% in the second year. Additionally, previous studies have suggested that greater bone loss occurs in the first year after surgery, with this loss stabilizing in subsequent years [30, 31]. Thus, the success rates of bone grafts should be compared according to periods of predetermined postoperative assessment periods. Moreover, the suggested postoperative follow-up time should cover a period of 6 months to 2 years [19, 30, 31].

Variables such as cleft type, laterality (unilateral or bilateral), cleft side, and donor area were not statistically associated with the efficacy of autogenous bone grafts, which is in agreement with previous studies [17, 19]. However, the dental age [19, 27] and pre- [17–19] or postgraft [4, 19] dental orthodontic therapy seem to influence the stability of bone grafts.

Although we did not assess the association between orthodontic treatment and secondary bone grafts, it is essential to make certain considerations. For example, the correction of posterior crossbites and alignment of the anterior teeth, as well as the association with the achievement of secondary bone grafts, seem to be the main concerns of orthodontic-surgical treatment in individuals with cleft in the alveolar region [35]. Constriction of the maxillary arch results from the approximation of the palatal processes, which are partly caused by the cleft itself but also may be accentuated after primary plastic surgeries [36]. Thus, although there is no consensus regarding the treatment protocol for individuals with CLP, in terms of the sequence and timing of appropriate rehabilitation procedures, transverse maxillary expansion prior to completion of the grafts is often advocated [17–19, 35]. In addition, evidence of a higher success rate of bone grafts performed prior to the maxilla expansion can be found in the literature [17–19]. However, da Silva Filho et al. [4] showed that the performance of rapid maxillary expansion, after alveolar bone grafting, was possible without jeopardizing the final results of the surgical procedure.

Additionally, when the canine erupts spontaneously, it creates a supportive and protective periodontium, which favors the height of the interdental bone septum [9, 14]. In contrast, when this does not occur, orthodontic traction is needed [5, 9, 12, 13]. In the present study, among the bone grafts performed before the eruption of the permanent canines, 44,8% erupted spontaneously through the graft after surgery, although the eruption rate was less than that reported in previous studies (ranging from 72% to 97%) [5, 13, 14, 28]. However, this rate was similar to those in other studies reporting values of 27% (Eldeeb et al., 1982) [9] and 41% (Eldeeb et al., 1986) [12]. These differences in the rate of eruption of the canine teeth adjacent to the cleft could be explained by the different observation periods after surgery. In our study, the results showed that longer follow-up periods for bone grafts were associated with a greater frequency of canines erupted. Additionally, although a significant association between the laterality of CLP and effectiveness of the bone grafts was not observed, greater number of canines were found to erupt after bone grafting in cases of unilateral clefts, although similar findings were not observed in earlier studies [5, 14, 28].

## Figures and Tables

**Table 1:** Characterization of the study sample, considering variables related to the population (*n* = 36; gender, age, type, and laterality of CLP) and the autogenous bone graft (*n* = 40; donor area, postoperative complications, and position of the permanent canine at the time surgery).

Sample characterization			Number	Frequency (%)
Population	Gender	Male	20	55.56
		Female	16	44.40
	Age	<10 years old	19	52.78
		11-12 years old	14	38.89
		≥13 years old	3	8.33
	Type of cleft	Cleft lip involving alveolar ridge (CL)	6	16.67
		Cleft lip and palate (CLP)	30	83.33
	Cleft laterality	Unilateral cleft (UC)	32	88.89
		Bilateral cleft (BC)	4	11.11

Bone grafts	Donor area	Iliac crest	32	80
		Intraoral region	8	20
	Postoperative complications	Infection	4	10
		Resorption	4	10
		Suture dehiscence of the graft	2	5
	Position of the permanent canine	Nonerupted	29	72.5
		Erupted	11	27.5

**Table 2:** Analysis of the agreement between the Bergland and Chelsea scales, considering the effectiveness of autogenous bone grafts with the Kendal rank correlation coefficient (*P* = 0.00).

		Chelsea scale		Total
		Satisfactory	Unsatisfactory	
Bergland scale	Satisfactory	24	**1**	25
	Unsatisfactory	0	4	4

Total		24	5	29

**Table 3:** Analysis of the association between the eruption of permanent canine teeth adjacent to the CLP (*n* = 13 erupted canines; *n* = 30 postgraft impacted canines) and the radiographic follow-up period, gender, and type and laterality of the CLP (Mann-Whitney *U* Test^*^; *α* ≤ 0.05).

Canine eruption		Number	Frequency (%)	Significance level
Radiographic follow-up period	≤6 months	0	0	**P** ** = 0.001** ^*^
	7–12 months	2/13	15.4	
	13–24 months	1/13	7.7	
	25–36 months	5/13	38.5	
	37–48 months	5/13	38.5	

Gender	Male	6/18	33.3	**P** ** = 0.046** ^*^
	Female	7/12	58.3	

Type of cleft	Incisive preforamen	2/6	33.3	*P* = 0.113
	Incisive transforamen	11/24	45.8	

Involvement side of cleft	Unilateral	12/22	54.5	**P** ** = 0.02** ^*^
	Bilateral	1/8	12.5	
